# Expression of human A53T alpha-synuclein in the rat substantia nigra using a novel AAV1/2 vector produces a rapidly evolving pathology with protein aggregation, dystrophic neurite architecture and nigrostriatal degeneration with potential to model the pathology of Parkinson's disease

**DOI:** 10.1186/1750-1326-5-43

**Published:** 2010-10-28

**Authors:** James B Koprich, Tom H Johnston, M Gabriela Reyes, Xuan Sun, Jonathan M Brotchie

**Affiliations:** 1 Toronto Western Research Institute, Toronto Western Hospital, University Health Network, 399 Bathurst Street, Toronto, ON, M5T 2S8, Canada

## Abstract

**Background:**

The pathological hallmarks of Parkinson's disease (PD) include the presence of alpha-synuclein (α-syn) rich Lewy bodies and neurites and the loss of dopaminergic (DA) neurons of the substantia nigra (SN). Animal models of PD based on viral vector-mediated over-expression of α-syn have been developed and show evidence of DA toxicity to varying degrees depending on the type of virus used, its concentration, and the serotype of vector employed. To date these models have been variable, difficult to reproduce, and slow in their evolution to achieve a desired phenotype, hindering their use as a model for testing novel therapeutics. To address these issues we have taken a novel vector in this context, that can be prepared in high titer and which possesses an ability to produce neuronally-directed expression, with expression dynamics optimised to provide a rapid rise in gene product expression. Thus, in the current study, we have used a high titer chimeric AAV1/2 vector, to express human A53T α-syn, an empty vector control (EV), or green fluorescent protein (GFP), the latter to control for the possibility that high levels of protein in themselves might contribute to damage.

**Results:**

We show that following a single 2 μl injection into the rat SN there is near complete coverage of the structure and expression of A53T α-syn or GFP appears throughout the striatum. Within 3 weeks of SN delivery of their respective vectors, aggregations of insoluble α-syn were observed in SN DA neurons. The numbers of DA neurons in the SN were significantly reduced by expression of A53T α-syn (52%), and to a lesser extent by GFP (24%), compared to EV controls (both *P *< 0.01). At the level of the striatum, AAV1/2-A53T α-syn injection produced dystrophic neurites and a significant reduction in tyrosine hydroxylase levels (by 53%, *P *< 0.01), this was not seen in the AAV1/2-GFP condition.

**Conclusions:**

In the current implementation of the model, we recapitulate the primary pathological hallmarks of PD, although a proportion of the SN damage may relate to general protein overload and may not be specific for A53T α-syn. Future studies will thus be required to optimise the dose of AAV1/2 employed before fully characterizing this model. The dynamics of the evolution of the pathology however, provide advantages over current models with respect to providing an initial screen to assess efficacy of novel treatments that might prevent/reverse α-syn aggregation.

## Background

A pathological feature of Parkinson's disease (PD), irrespective of etiology, is the presence of intraneuronal Lewy bodies and Lewy neurites. The appearance of Lewy bodies in different brain regions parallels the development of symptom severity [1-3]. Lewy bodies are composed of at least 45 identified proteins and lipids, although the most significant contribution is made by α-synuclein (α-syn) [4-8]. Loss of dopamine (DA) neurons and the presence of Lewy bodies in the substantia nigra (SN) together provide the definitive diagnosis of PD [9]. The precise role for α-syn in the CNS, however, has yet to be determined. α-syn binds readily to lipids [10] and appears to be associated with vesicles in the presynaptic terminal [11-13] suggesting that the physiological role of α-syn might be related to vesicular release at the lipid membrane.

Human familial and sporadic cases of PD and animal models demonstrate that, in some form, α-syn contributes to the development of PD. Thus, familial forms of PD can be caused by mutations of α-syn (A53T, A30P, and E46K)[14-16] as well as by duplication and triplication of the wildtype (Wt) allele, *SNCA *[17-19]. Post-mortem analyses of sporadic forms of PD reveal Lewy bodies heavily stained for α-syn, although the mechanism of their production is unclear. Less convincing, perhaps, though still supportive of a role for α-syn in dopaminergic dysfunction in PD, are transgenic mice over-expressing α-syn. These animals show motor abnormalities and impairment in striatal DA release, though no overt loss of DA neurons [13,20,21]. Over-expression of α-syn (whether Wt or mutated), through the use of viral vectors, in the rodent [22-25] and primate [26,27] SN, has provided a more clear indication of toxicity, with DA neuron loss in the SN, dystrophic neurites, reductions in striatal DA, and motor behaviour impairments. Taken together, these data all support the hypothesis that α-syn is toxic to DA neurons.

In normal human, α-syn is evenly distributed throughout the neuropil [28]. In PD, however, staining in surviving dopamine neurons is largely in clumps, or aggregates, that are seen throughout the soma. A large proportion of these α-syn aggregates are resistant to protein digestion techniques and considered to be insoluble [29-32]. It is tempting to speculate that aggregation of α-syn is a toxic process in PD and thus, that solubilising, or de-aggregating would be an effective approach to disease modification. However, using post-mortem tissue, it is difficult to discern whether the neurons that have already died in PD had α-syn aggregates similar to those that have survived. Animal models of synucleinopathy are thus important to critically assess the relevance of targeting α-syn aggregation as a therapy for PD. While the gene delivery based animal models of synucleinopathy, have proved better than transgenic models, in so far as reproducing overt nigrostriatal damage, limitations exist. Thus, to date, none of the viral vector models, whether based upon adeno-associated viral vectors (AAV) or lentiviruses have proved optimal for addressing these questions or providing a reliable platform upon which drug discovery programmes might usefully build. Thus, in some cases the timeframe for development of damage is long (8-16 weeks) [22,23,25], and in others there is high variability within studies [24,27]. We propose that these limitations are purely technical, relating to the specifics of the virus and that a delivery vector, with a strong promoter, and selective neuronal expression, if produced in high titer could produce a model of PD synucleinopathy that would have even greater utility. Our preferred model would have the following properties: (1) high levels of expression of α-syn in SN and striatum, after SN delivery (2) pathology that developed over a time period that allowed investigation of effects of manipulations initiated before and after α-syn damage had occurred (3) relatively rapid evolution of the model, 3-6 wks, to minimise logistical challenges in running studies with the model (4) an evolving behavioural deficit (5) pathology that had characteristics similar to Lewy bodies and neurites e.g. intraneuronal inclusions of α-syn in the SN and dystrophic neurites in the striatum and (6) damage that was specific for α-syn and not purely a result of general over-expression of a protein.

As a first step towards achieving these goals we have developed a novel AVV model of PD alpha synucleinopathy. This vector is based on combining the distinct advantages of serotype 2 (neuronal tropism, ability to produce high titers, and to purify on a heparin sulphate column) with that of serotype 1 (excellent ability to penetrate brain tissue), using a chimeric approach to its construction. Thus, AAV1/2 is a vector that expresses both AAV serotype 1 and 2 on the viral capsid in a one to one ratio (details in methods section).

In the current study we have used this AAV1/2 vector to produce a rat model of PD based on the targeted expression of human A53T α-syn in the SN and using GFP and an empty vector as controls. The principle aim of this study was to provide an initial assessment of whether these vectors can drive expression in neurons of the substantia nigra, whether that protein is transported to terminals in the striatum and whether such expression is associated with aggregate-like pathology and loss of dopaminergic phenotype. To this end we report that high titer AAV1/2 vectors produce a relatively rapid course (3 weeks) of dopaminergic nigrostriatal pathology in the presence of α-syn aggregates and dystrophic axonal morphology and that GFP also shows evidence of toxicity.

## Results

### Expression of GFP and human A53T alpha-synuclein along the nigrostriatal path

**Figure 1:**
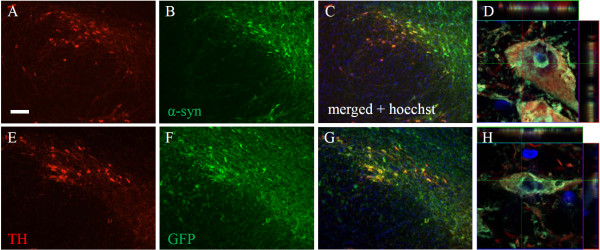
**Colocalization of tyrosine hydroxylase and alpha-synuclein or GFP in the substantia nigra**. Immunofluorescent staining of tyrosine hydroxylase (A, E), GFP (B), and human alpha-synuclein (F) 3 weeks following AAV1/2 injections. Panels C and G represent merged images of A + B and E + F, respectively. Panels D and H represent high power confocal images revealing aggregates of human  alpha-synuclein and GFP within a TH neuron of the substantia nigra and confirmed by the orthogonal view of each panel. Scale bar is 500 μm.

Delivery of AAV1/2-A53T alpha-synuclein to the SN of rats produced widespread expression in TH-immunoreactive neurons across the entire rostral-caudal axis of the SN. Of the SN neurons still expressing TH, the vast majority of them co-localized with human α-syn (Figure 1G). Co-localization of TH and α-syn within a single neuron was confirmed by high magnification confocal imaging and after review of z-stacks (Figure 1H). Aggregation of alpha-synuclein within the cytoplasm of TH-immunoreactive neurons is shown in Figure 1H and could be seen in most α-syn/TH positive cells.

Expression of GFP following delivery of AAV1/2-GFP to the SN was also shown to co-localize with TH-immunoreactive neurons (confirmed with z-stacks) throughout the SN (Figure 1C, D). GFP aggregates were observed in the majority of cells within the SN that expressed TH (Figure 1D).

**Figure 2:**
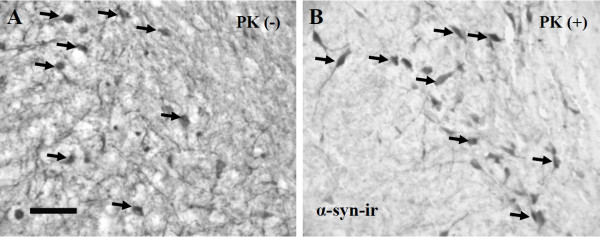
**Alpha-synuclein deposits in nigral neurons are insoluble aggregates**. Midbrain sections from rats injected with AAV1/2 A53T α-syn were treated with proteinase K (B, PK+) or the buffer used to dissolve PK (A, PK^-^) and subsequently immunostained for human α-syn to determine its solubility. Panel A (PK^-^) shows that α-syn expression fills the substantia nigra expressing in both nigral neurons and neurites. In panel B, following PK digestion, many neurons maintain expression of α-syn aggregates (insoluble α-syn), while the neurites appear to have been largely cleared (soluble α-syn). Arrows point to examples of α-syn filled neurons. Scale bar in panel A is 200 μm.

A pathological feature of PD is that a significant proportion of α-syn inclusions in nigral neurons are considered to be aggregated (insoluble inclusions) [5,7,28]. In order to evaluate the solubility of the α-syn deposits seen in this model we conducted a proteinase K (PK) digestion on midbrain sections from AAV1/2 A53T α-syn rats. We found that the majority α-syn inclusions in our model were resistant to PK digestion and could thus be considered insoluble aggregates (Figure 2).

**Figure 3:**
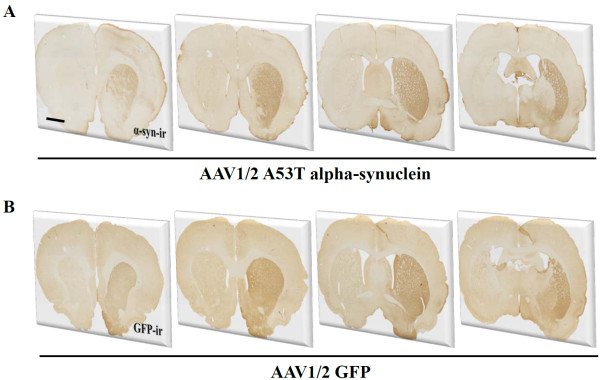
**Expression of transgenes in the striatum**. Both GFP and human alpha-synuclein were widely expressed throughout the striatum 3 weeks following delivery of AAV1/2 to the substantia nigra. Scale bar in panel A is 500 μm.

Transport of viral vector mediated GFP or alpha-synuclein along the nigrostriatal projection was indicated by expression in terminals throughout the striatum. Figure 3A, B shows that both GFP and alpha-synuclein were transported along axons from neurons of the SN and terminating in the striatum. Expression was observed from the most anterior to the most posterior extent of the striatum.

### Nigral delivery of AAV1/2-A53T alpha-synuclein and AAV1/2-GFP produce neuronal loss in the SN

**Figure 4:**
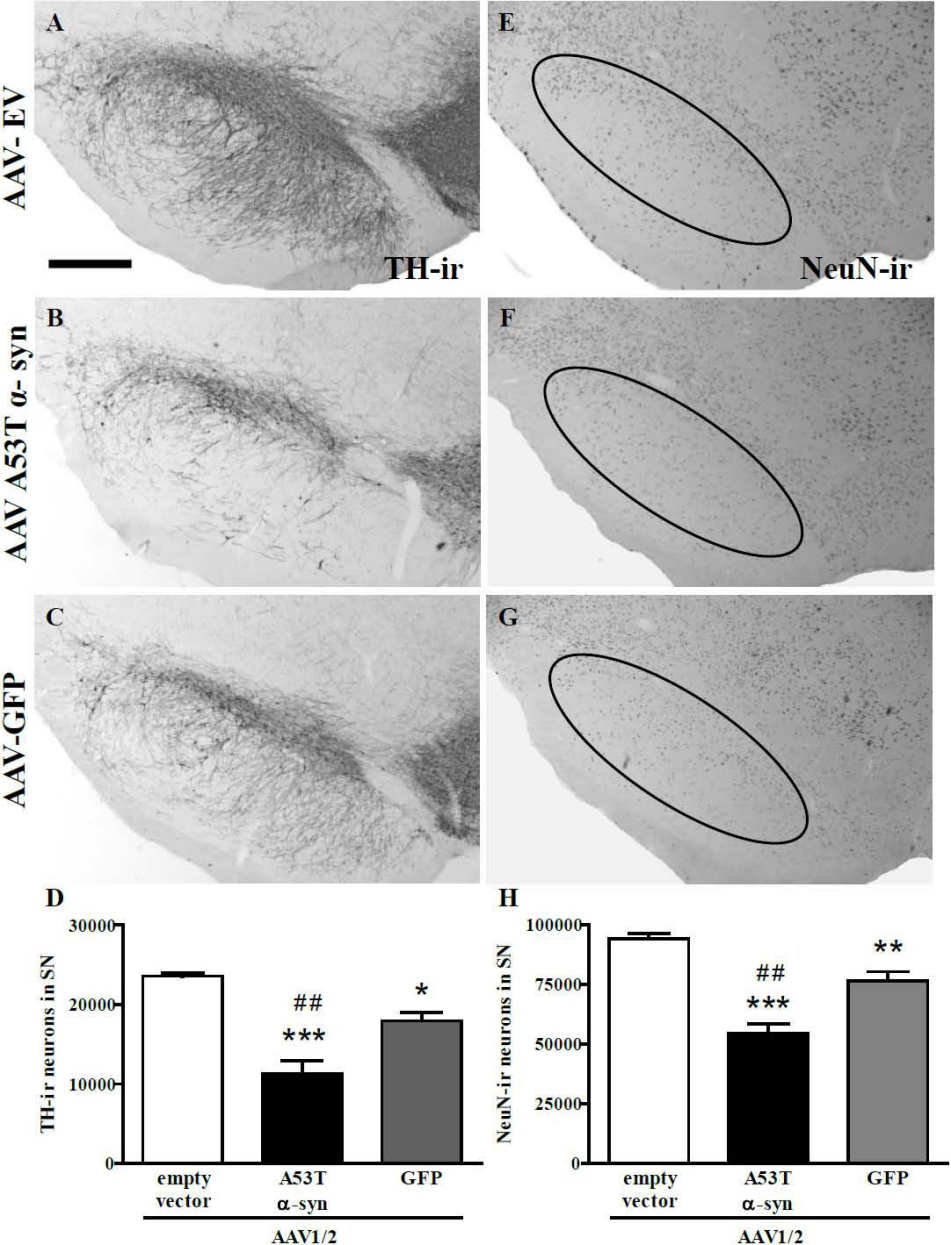
**Tyrosine hydroxylase and NeuN cell counting in the substantia nigra**. Three weeks following delivery of AAV1/2 to the substantia nigra there is significant reductions in the number of TH-immunoreactive neurons and NeuN-immunoreactive neurons for both alpha-synuclein and GFP treated animals compared to empty vector controls. AAV1/2 A53T alpha-synuclein produced significantly greater TH and NeuN cell loss compared to the GFP group. *P < 0.05 *cf*. empty vector; ** *P *< 0.01 *cf*. empty vector;*** *P *< 0.001 *cf*. empty vector; ^## ^*P *< 0.01 *cf*. GFP; scale bar is 1000 μm.

Three weeks following surgical delivery of AAV1/2 vectors to the rat SN there were significant differences in the amount of TH-immunoreactive neurons present quantified using unbiased stereology [F(2,12) = 26.15, *P *< 0.0001] (Figure 4A-D). To serve as a control, identical AAV1/2-empty vectors were delivered in the same manner and concentration. *Post-hoc *analysis revealed that rats that received AAV1/2-A53T α-syn had significantly less TH-immunoreactive neurons compared to those injected with either AAV1/2-empty vector (52% reduction, *P *< 0.001) and GFP (37% reduction, *P *< 0.01). Furthermore, there were significantly less TH-immunoreactive neurons in rats receiving AAV1/2-GFP compared to those that received AAV1/2-empty vector controls (24% reduction, *P *< 0.05). To confirm that the AAV1/2-EV was not toxic to DA neurons we counted TH-immunoreactive neurons in the SN opposite to the injected side and showed that there was no significant difference between hemispheres (t[8] = 1.09, *P *> 0.05).

Further analysis was conducted to determine whether viral vector mediated reductions in TH-immunoreactive neurons of the SN were due to a loss in phenotypic expression or neuronal death. To address this we performed stereological cell counts in the SN that was immunohistochemically labelled with a universal neuron marker, NeuN. The numbers of NeuN-immunoreactive cells were significantly different across treatment groups [F(2,12) = 33.29, *P *< 0.0001] (Figure 4E-H). Delivery with AAV1/2-A53T alpha-synuclein produced a 42% and a 29% decrease in NeuN-immunoreactive cells compared to AAV1/2-empty vector (*P *< 0.01) and AAV1/2-GFP (*P *< 0.01) treated groups respectively. Animals receiving AAV1/2-GFP showed a significant reduction in NeuN-immunoreactive cells compared to AAV1/2-EV controls (19% reduction, *P *< 0.01).

### Expression of A53T alpha synuclein, but not GFP, reduces tyrosine hydroxylase expression in the striatum

**Figure 5:**
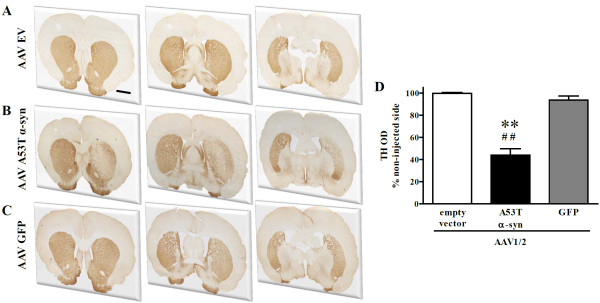
**Tyrosine hydroxylase immunostaining in the striatum**. AAV1/2 human A53T alpha-synuclein produced significant reductions in the optical density measurements of tyrosine hydroxylase (TH) compared to empty vector and GFP treated groups 3 weeks following AAV1/2 injection into the substantia nigra. **P < 0.01 *c.f*. empty vector; ^##^*P *< 0.01 *c.f*. GFP. Scale bar in panel A is 500 μm.

In the striatum, the degree of TH expression was assessed using optical density (OD) measurements of immuno-labelled tissue. Three anatomical levels were assessed (pre-commissural, commissural, and post-commissural; see Figure 5) and for each level the contralateral striatum was also assessed. Final values represent the average of the 3 anatomical levels taken as a percentage of the average of each corresponding contralateral level. There were significant differences in TH-OD across groups [F(2,13) = 18.57, *P *< 0.001] (Figure 5C). Animals injected with AAV1/2-A53T α-syn showed significant reductions in TH-OD compared to AAV1/2-EV (*P *< 0.01) and AAV-1/2-GFP treated groups (*P *< 0.01), while no change in TH-OD was observed in animals that received AAV1/2 GFP compared to EV controls (*P *> 0.05, NS) (Figure 5).

To see whether the observed reduction in TH expression was maintained, we analyzed tissues that had been exposed to AAV1/2-A53T α-syn for an additional 3 weeks. Thus, 6 weeks following injection of AAV1/2-A53T α-syn we found that striatal TH remained significantly reduced (t[8] = 2.57, *P *< 0.05; 167 ± 21 vs. 223 ± 4 [mean optical density ± SEM]) compared to its respective non-injected side. Further analysis of dopaminergic fibres was conducted using dopamine transporter (DAT) as a marker to support the apparent loss of nigrostriatal projections at this timepoint. We found that striatal DAT levels (nCi/g) were also significantly reduced (t[8] = 2.81, *P *< 0.05; 287 ± 65 vs. 483 ± 24 [mean optical density ± SEM]) compared to its respective non-injected side.

### Dystrophic axonal morphology is present in the striatum of AAV1/2 A53T alpha-synuclein injected rats

**Figure 6:**
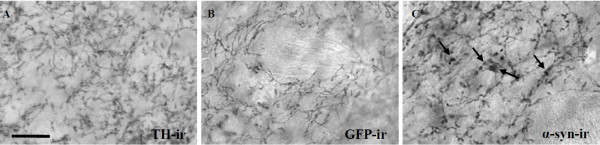
**Axon morphology in the striatum of AAV1/2 treated rats**. Delivery of AAV1/2 human A53T alpha-synuclein to the substantia nigra produces dystrophic and bulging neurites in the striatum 3 weeks later. These changes in axonal morphology do not appear in the AAV1/2 GFP (GFP-immunoreactive [-ir]) or empty vector (represented by tyrosine hydroxylase-immunoreactive [ir]) treated animals. Arrows point to evidence of human alpha-synuclein positive dystrophic neurites; scale bar is 50 μm.

Dystrophic and swollen neurites, labelled with a human specific α-syn antibody, were prevalent throughout the striatum of AAV1/2-A53T α-syn injected animals (Figure 6A). Similar morphology was observed in these same animals using TH-immunoreactive, although, given the significant decrease in TH optical density in this region (Figure 5), less fibres were strongly labelled (data not shown). No abnormal morphological features were seen in GFP-immunoreactive striatal tissue and fibres appeared similar to TH-immunoreactive fibres of the AAV1/2-EV treated animals (Figure 6B, C).

## Discussion

The objective of the current study was to develop a rat model of Parkinson's disease based on the targeted over expression of human A53T α-syn that produced pathology in a time frame amenable to initial *in vivo *evaluation of potential therapeutics. We show that within 3 weeks of injection of high titer AAV1/2 viral vectors (5.1 × 10^12 ^gp/ml) into the SN there are significant signs of dopaminergic toxicity in the nigrostriatal pathway.

Delivery of a single 2 μl injection of AAV1/2 A53T α-syn to the rat SN produced excellent anatomical coverage of the target region, the SN. Thus, α-syn expression was observed in the vast majority of TH-immunoreactive neurons spanning the anterior-posterior and dorso-ventral boundaries of the SN. Transport of α-syn and GFP by DA neurons of the SN was determined by examining fibre staining in the primary target structure, the striatum. Indeed, the entire striatum was filled with human α-syn or GFP stained fibres, indicating that A35T α-syn and GFP are able to travel along the nigrostriatal path and confirms that the majority of SN TH-immunoreactive neurons remaining are producing the vector mediated proteins. Thus, with respect to coverage of SN DA neurons and their axons projecting to the striatum, the AAV1/2 construct utilized in this experiment was highly effective. It is likely that the properties of the serotype 1 component of our construct and the high titer that could be purified afforded the AAV1/2 its ability to penetrate brain tissue with such efficacy [33,34]. In a study by McFarland et al (2009), several AAV serotypes of similar concentration were compared, and serotype 1 had superior coverage of expression at the level of the SN compared to others (serotypes 2, 5, and 8) [33]. Use of the AAV2/5 construct with titers greater than those used here (6.2 - 6.7 × 10^13 ^gc/ml) has also produced coverage of the SN in similar magnitudes using only a single 2 μl injection into the SN [35] and may provide an alternative means of producing models with excellent anatomical coverage, though the impact of such high titers on non-specific damage remains to be evaluated (see below).

Three weeks after delivery of AAV1/2-A53T α-syn, aggregates of human α-syn were observed in nigral TH-immunoreactive cells. Aggregates of α-syn were determined to be insoluble as indicated by being resistant to proteinase K digestion, a cardinal feature observed in PD [7,28,29,32]. There was a reduced number of nigral TH-immunoreactive neurons, as well as reduction in total neuronal (NeuN) number, and, within the striatum, a loss of TH-immunoreactive fibre density and the presence of α-syn/TH-positive dystrophic neurites. Furthermore, this reduction in TH fibre density was present through 6 weeks with a concomitant reduction in striatal DAT demonstrating a persistence of effect. Thus, the approach of AAV1/2 delivery of A53T α-syn was able to replicate, in only three weeks, the major pathological hallmarks of PD. The model used here is not the first AAV-based delivery of α-syn to replicate α-syn aggregation, dystrophic neurites, and similar patterns of nigral cell loss. This has been previously demonstrated in other AAV α-syn rat models [22,23,25,35], although with longer durations to achieve endpoints (8-16 weeks) and variable degrees of cell loss (32-50%). However, the magnitude of the losses (53% reduction in SN DA neurons), the low variability (n = 6/group) and the short time course of development (3 weeks) of the pathologies seen in the current study, highlight some of the potential advantages of using a high titer AAV1/2 vector when considering a model for assessing potential therapeutics targeting the toxicity of α-syn in PD.

AAV1/2 delivery of GFP was employed to control for the effects of high expression of any exogenous protein. This control demonstrated that the high titer of AAV1/2-GFP employed here caused significant nigral dopaminergic toxicity and thus should be considered when interpreting the toxic effects produced by delivery of AAV1/2 α-syn or other high titer vectors. The toxicity of AAV1/2-GFP however, could not account for all the A53T α-syn induced damage in the nigra, being significantly less, and being limited to the SN (no loss in striatal TH-immunoreactive or signs of dystrophic neurites). Thus, three weeks following the delivery of AAVs, aggregates of both human α-syn and GFP were observed in TH-immunoreactive neurons of the SN. Aggregates were seen in the majority of cells expressing both TH and α-syn or GFP. Unbiased stereology demonstrated that AAV1/2-A53T α-syn and, to a lesser extent, AAV1/2-GFP resulted in significant reductions in TH-immunoreactive neurons compared to AAV1/2-EV injected rats. In a recent study by Sanchez-Guajardo et al. (2010) AAV2/5 was used to deliver human α-syn (6.2 × 10^12 ^gp/ml) or GFP (7.9 × 10^12 ^gp/ml) as a control. The results of that study showed that GFP and α-syn expression produced ~20% and 50% SN DA neuron loss, respectively. These data support those shown here, in that expression of a control protein can be toxic and needs to be considered in the interpreation of the results.

To determine whether the TH-immunoreactive cell loss in the SN of the current study was indicative of a down-regulation of TH or frank neuronal cell loss, stereological assessment of NeuN-positive cells was performed [22,36,37]. NeuN cell counts were also significantly reduced in AAV1/2-A53T α-syn and AAV1/2-GFP injected animals when compared to AAV1/2-EV controls. In both cases (TH and NeuN cell number), the rats that received AAV1/2-A53T α-syn had significantly greater cell loss than AAV1/2-GFP administered animals. The toxicity of AAV1/2-GFP is unlikely to result from toxicity due to the vector itself, as, in so far as the dependent measures used in this study, injection of AAV1/2-EV produced no evidence of degeneration, in either the SN or the striatum. This suggests that overloading of the control protein might, in itself, be the cause of some of the SN damage seen with AAV1/2-GFP used at this titer. Indeed, a similar effect of GFP toxicity to SN DA neurons has been reported with the use of AAV8 (4 μl of 1 × 10^10 ^vector genomes/ml) four weeks following injection [38]. Burdening the DA neurons of the SN with excessive protein likely reduces their fitness and leads to their demise. Cellular systems, lysosomal and ubiquitin proteasome, designed to deal with protein turnover have been shown to be significantly more active in DA neurons following viral vector mediated expression of α-syn and also in PD patients [29], although to no avail, indicating that insufficient clearance of unwanted or misfolded proteins may pose a significant threat. These findings highlight the importance of not only EV controls, which are often used, but also control protein vectors to define whether generalised protein overload underlies damage attributed to purported toxic proteins such as α-syn.

However, while there may be a non-α-syn-specific component to SN damage caused by delivery of AAV1/2-A53T α-syn, the striatal damage, loss of TH and dystrophic neurite pathology, appears to be specific to the expression of A53T α-syn. Thus, if this high titer AAV1/2 model were to be used, for instance to evaluate novel therapeutic strategies targeting α-syn, it might be appropriate to concentrate on striatal endpoints. There currently exist several classes of drugs that could be used in this initial screen that have been shown to reduce α-syn levels *in vivo*. For example, one of the statin drugs, lovastatin, was reported to reduce α-syn levels in the cortex of transgenic mice that overexpress α-syn [39]; rifampicin (a bacterialcidal antibiotic) has been shown to reduce established α-syn aggregate load and associated insoluble oligomers in a mouse model of multiple system atrophy that resulted in a decrease in neurodegeneration [40]; and finally rapamycin, a macrolytic lactone, that has been shown to reduce α-syn levels ostensibly via activation of autophagy [41,42]. However, in future implementations of this model, it may be useful to evaluate lower titers of AAV1/2 to define if such a dose can be identified which will produce SN toxicity that can be completely ascribed to A53T α-syn delivery and have no component that might be caused by generalised protein overload.

## Conclusions

Clinical and experimental evidence points towards the presence of α-syn aggregates in catecholaminergic neurons in PD as a contributing factor to reduced neuronal health and ultimately cell death. Irrespective of etiology, the vast majority of PD cases receiving post-mortem evaluation show Lewy bodies and Lewy neurites which stain heavily for α-syn [5]. This suggests that of the multiple upstream mechanisms, whether of genetic and/or non-genetic origin, responsible for PD, there appears to be convergence that leads to synucleinopathy. Such convergence defines α-syn as a key intervention point at which therapeutics could broadly target PD. It remains unclear whether the end product of α-syn accumulation, the aggregated form in Lewy bodies, is toxic or whether species leading up to this form should be the focus of therapeutic strategies. *In vivo *models such as that described here will prove useful in understanding which types of therapies will be effective by providing a relatively rapid screening platform upon which to test drugs capable of preventing aggregate formation or by reversing/de-aggregating inclusions. Care must be taken however, in interpreting actions where the damage cannot be completely ascribed to the α-syn per se, where there might be some role of generalised increased demand on protein handling systems. Indeed, more work is needed to refine models that do not generate toxicity in the control protein condition. This will likely require lower vector concentrations and a somewhat longer time course. There may always be a balance between non-specific toxicity and duration of evolution of pathology. However, we could envisage an ideal model based around the current AAV1/2 technology where pathology and behavioural deficits, selective for α-syn over generalised over-expression of protein, evolved over a relatively short period of time, perhaps 6 weeks, but where there was an intermediate stage, perhaps at 3 weeks, where there was functional disruption of DA systems though in the absence of overt cell loss. Treatments that were deemed efficacious in the rapid onset model, such as that described here, could then be further evaluated in a more protracted or progressive model to define in greater detail their therapeutic potential.

## Methods

### Animals and vector delivery

24 female Sprague Dawley rats (~280 g, Charles River) were stereotaxically injected with either AAV1/2 empty vector, GFP, or human A53T alpha-synuclein under isoflurane/oxygen anesthesia. In each case, a single 2 μl injection of viral vector was delivered to the SN at a rate of 0.2 μl/min according to the following coordinates: AP, -5.2 mm; ML, -2.0 mm; DV, -7.5 mm (skull) using a microinjector (Stoelting, Kiel, WI) and according to the atlas of Paxinos and Watson (1997). Animals were housed in pairs in a temperature controlled environment, kept on regular 12 hr light/dark cycle and allowed food and water *ad libitum*. All procedures were conducted under a permit that had received local IACUC approval (University Health Network).

### Vectors

Adeno associated vectors were custom ordered from GeneDetect Ltd. (Auckland, New Zealand). Each vector was driven by a chicken beta actin (CBA) promoter hybridized with the cytomegalovirus (CMV) immediate early enhancer sequence. In addition, a woodchuck post-transcriptional regulatory element (WPRE) and the presence of a bovine growth hormone (BGH) polyadenylation sequence for high transcription following transduction was incorporated. Viral titers were 5.1 × 10^12 ^genomic particles (gp)/ml for each vector used and were determined by quantitative PCR (Applied Biosystems 7900 QPCR) with primers directed to the WPRE region incorporated within the AAV expression cassette, thus representing the number of functional physical particles of AAV in the solution containing the genome to be delivered. WPRE primers used produced a 113 bp product and were as follows (5' to 3'): FWD GGCTGTTGGGCACTGACAAT; REV CCGAAGGGACGTAGCAGAAG. AAV1/2 is a chimeric vector where the capsid expresses AAV1 and AAV2 serotype proteins in a 1:1 ratio and use the inverted terminal repeats (ITR's) from AAV2 according to the following scheme: ITR (from AAV2)----CMV/CBA promoter----human A53T alpha-synuclein or GFP----WPRE-bGH-polyA----ITR (from AAV2) [43]. Viral vectors were affinity purified against immobilized heparin sulphate proteoglycan and concentrated by modified Iodixanol/cation exchange/Q-Sepharose. Purity was verified by SDS-PAGE. Three vectors were produced using this design, in the first, the gene for mutated human alpha-synuclein was inserted, this vector is termed AAV1/2-A53T α-syn, the second vector expressed green fluorescent protein (GFP), AAV1/2-GFP, and in the third vector the space for the gene was left empty, AAV1/2-empty vector (EV). Similar AAV1/2 chimeric vectors have previously been used for other purposes i.e. expressing proteins in experimental models of Huntington's disease and epilepsy [34,44].

#### Post-mortem measures

##### Immunohistochemistry

Three weeks after AAV1/2 injection, animals were administered an overdose of pentobarbital and killed by exsanguination with saline followed by 4% paraformaldehyde, brains removed and processed for immuno-labeling. Brains were sectioned frozen in the coronal plane at a thickness of 40 μm on a sliding microtome (Leica Microsystems Inc., Richmond Hill, ON) and 6 series of sections were stored in cryoprotectant (30% glycerol, 30% ethoxyethanol, 40% PBS). A single series of sections were processed for visualization of tyrosine hydroxylase (TH) and NeuN via the biotin-labelled antibody procedure. Briefly, following several washes in a PBS solution containing 0.2% Triton X-100 (PBS-T), endogenous peroxidase was quenched in a 3% hydrogen peroxide solution and background staining was then inhibited in a 10% normal goat serum/2% bovine serum albumin solution. Tissue was then incubated with primary antibodies overnight: rabbit anti-TH antibody (1:1000, Pel-Freez, Rogers, AR), mouse anti-NeuN antibody (1:200, Millipore, Billerica, MA). After three washes in PBS-T, sections were sequentially incubated in biotinylated goat anti-rabbit or mouse IgG (1:300; Vector, Burlingame, CA) for 1 h and the Elite avidin-biotin complex (ABC Kits; Vector, Burlingame, CA) for 1 h separated by three washes in PBS. Immunostaining was visualized following a reaction with 3,3-diaminobenzidine (Vector, Burlingame, CA). Sections were then mounted on glass slides, allowed to dry, dipped into dH_2_0, dehydrated through graded alcohols (70%, 95%, 100%), cleared in xylenes, and coverslipped with DPX mounting medium (Electron Microscopy Sciences, Hatfield, PA).

Triple label immunofluorescence (Confocal microscopy, Zeiss Axioplan with LSM510META) to reveal TH (1:1000, Pel-Freez, Rogers, AR) and human alpha-synuclein (1:500, Zymed, San Fransico, CA) or GFP (1:2000, Abcam, Cambridge, MA), and Hoechst (nuclear stain) simultaneously to provide detail regarding co-localization and whether expression was nuclear and/or cytoplasmic. Images were taken throughout the Z-axis to confirm co-localization of α-syn and GFP within individual TH neurons.

##### Stereology

TH and NeuN stained sections of the SN were used for stereological estimation of dopamine neuron numbers using optical fractionator from the Stereo Investigator software package (v. 7, MBF Biosciences, Williston, VT). The user was blinded to group assignment by coded slides. Nine sections spanning the entire anterior/posterior extent of the SN, separated by 240 μm (1/6 series), were used for counting. All TH-immunoreactive neurons of the SN (pars compacta and pars reticulata) were included within each contour, of each section. NeuN contours were closely matched with cases used for TH [22]. Parameters used for TH stereological counting were grid size, 300 μm × 300 μm; counting frame, 80 μm × 80 μm, and 2 μm guard zones. Parameters used for NeuN stereological counting were grid size, 480 μm × 480 μm; counting frame, 80 μm × 80 μm, and 2 μm guard zones. Tissue thickness was determined by the user at each counting site. All final values represent *estimated total by number weighted section thickness *and were only included if their Gunderson coefficient of error (m = 1) was less than 0.09.

##### Proteinase K treatment

Tissue sections from AAV1/2-A53T α-syn rats containing the SN were treated with proteinase K to determine whether the α-syn seen here was soluble (non-aggregated) or insoluble (aggregated) according to the method of Chu et al (2007 and 2009) [28,29]. Briefly, sections were mounted and dried on Permafrost^+ ^glass slides for at least 8 hrs at 55°C. Sections were then briefly hydrated with TBS-T (10 mM Tris-HCl, pH 7.8; 100 mM NaCl; 0.05% Tween20), and digested with 50 μg/ml PK (Invitrogen, Carlsbad, CA) in TBS-T (10 mM Tris-HCl, pH 7.8; 100 mM NaCl; 0.1% Tween20) for a period of 2.5 h at 55°C. Sections were then fixed for 10 min using 4% paraformaldehyde and then processed for α-syn immunohistochemistry as described above.

##### Autoradiography

The levels of striatal DAT binding were assessed by [^125^I]-RTI-121 binding autoradiography in sections prepared from fresh-frozen tissue. Briefly, thawed slides (one slide per animal) were placed in binding buffer (2 × 15 min, room temperature) containing 50 mM Tris, 120 mM NaCl and 5 mM KCl. Sections were then placed in the same buffer containing 30 pM [^125^I]-RTI-121 (Perkin-Elmer, specific activity 2200 Ci/μmol) for 120 min at 25°C to determine total binding. Non-specific binding was defined as that observed in the presence of 100 μM GBR 12909 (Tocris Bioscience). All slides were then washed (4 × 15 min) in ice-cold binding buffer, rinsed in ice-cold distilled water and air-dried. Together with [^125^I]-microscale standards (Amersham) slides were then apposed to autoradiographic film (Kodak) and left for 7 days at RT before developing. Autoradiograms were analysed using MCID software (Image Research Inc, Ontario, Canada). Densiometric analysis of 3 striata from each animal was carried out whereby a reference curve of c.p.m. versus optical density was calculated from β-emitting [^14^C] micro-scale standards and used to quantify the intensity of signal as nCi/g. Background intensity was subtracted from each reading. Data were then expressed as mean ± s.e.m. signal intensity for each treatment group. Non-specific binding was calculated in the same way and subtracted from the total to give to give specific binding. Non-specific binding was found to account for <1% of total binding.

#### Statistical Analysis

For all statistical comparisons we first used a 1-way ANOVA, with significance set at *P *< 0.05. If ANOVA was significant, all *post-hoc *tests were conducted using Tukey's Multiple Comparison test. Where only two groups were compared, an unpaired t-test was conducted. Software used to conduct statistical analyses and graph all data was Prism v. 5.02 (GraphPad, La Jolla, CA, USA).

## Competing interests

JBK and THJ have received consultancy fees from Atuka Ltd. JMB has received consultancy fees from, and holds an equity position in, Atuka Ltd. There are no conflicts of interest.

## Authors' contributions

JK, TH, and JMB conceived and designed the experiments and drafted the manuscript. MGR and XS contributed to the histology and cell counting components of the study. JK and TH performed the stereotaxic surgeries. JK conducted the statistical analyses and produced the figures. All authors read and approved the final manuscript.
